# Fluorescence-Free Biosensor Methods in Detection of Food Pathogens with a Special Focus on *Listeria monocytogenes*

**DOI:** 10.3390/bios7040063

**Published:** 2017-12-20

**Authors:** Rajeswaran Radhakrishnan, Palmiro Poltronieri

**Affiliations:** 1 Faraday Technology Inc., Englewood, OH 45315, USA; Rajeswaran@faradaytechnology.com; 2 ISPA-CNR, National Research Council, 73100 Lecce, Italy

**Keywords:** *Listeria monocytogenes*, fluorescencel-free biosensors, surface plasmon resonance (SPR), rlectrochemical impedance spectroscopy (EIS)

## Abstract

Food pathogens contaminate food products that allow their growth on the shelf and also under refrigerated conditions. Therefore, it is of utmost importance to lower the limit of detection (LOD) of the method used and to obtain the results within hours to few days. Biosensor methods exploit the available technologies to individuate and provide an approximate quantification of the bacteria present in a sample. The main bottleneck of these methods depends on the aspecific binding to the surfaces and on a change in sensitivity when bacteria are in a complex food matrix with respect to bacteria in a liquid food sample. In this review, we introduce surface plasmon resonance (SPR), new advancements in SPR techniques, and electrochemical impedance spectroscopy (EIS), as fluorescence-free biosensing technologies for detection of *L. monocytogenes* in foods. The application of the two methods has facilitated *L. monocytogenes* detection with LOD of 1 log CFU/mL. Further advancements are envisaged through the combination of biosensor methods with immunoseparation of bacteria from larger volumes, application of lab-on-chip technologies, and EIS sensing methods for multiplex pathogen detection. Validation efforts are being conducted to demonstrate the robustness of detection, reproducibility and variability in multi-site installations.

## 1. Introduction

Food pathogens, either anaerobic, microaerophilic or aerobic species, contaminate food processing plant surfaces or originate from food sources. Therefore, a limit has been fixed for the presence of colony forming units of pathogens. *Listeria monocytogenes* can contaminate a wide range of foods, including yogurt, cheeses, meat, ham, smoked salmon, poultry, seafood and vegetable products, especially their surfaces. Its presence poses a real threat to ready-to-eat foods, since the bacteria can survive and proliferate in adverse environmental conditions during food production and storage (such as low pH, refrigerated temperatures and high salt concentration).

International regulations for ready-to-eat foods ask for corrective actions in the presence of *L. monocytogenes:* the bacteria count should be lower than 0.04 CFU g^−1^ for food that supports the growth of the microorganism, and 100 CFU·g^−1^ for food not supporting the survival. It is necessary to determinate absence of *L. monocytogenes* in foods destined to infants [1,2,3,4]. Microbiological criteria for *L. monocytogenes* in food safety are based on microbiology laboratory culture methods. *L. monocytogenes* detection methods, i.e., UNI EN ISO 11290-1:2005, include a pre-enrichment step of a food sampling followed by growth on selective solid medium: the protocol takes four days for completion. Molecular methods have taken the lead in pathogen detection, thanks to shorter times of handling, with results obtained in a few hours. Real time PCR is the main molecular method applied in food analysis: Recently, highly reliable diagnostic kits, such as the AFNOR validated iQCheck, allowed to limit the pre-enrichment step to 18 h, eliminating one day of culture, combined with reliable real time PCR analysis kits [3,4,5]. 

Biosensors have been applied to pathogen detection in liquid and solid food samples, but only in few cases a low limit of detection (LOD) in the range of the LOD achieved by PCR has been obtained. Recently, performing immunomagnetic separation on pre-enrichment cultures of *L. monocytogenes*, combined with fluorescent antibodies and detection on protein chips, the methods obtained good sensitivity with values reaching the sensitivity of PCR analysis [2,3]. Therefore, it is the interest of food industry players to see novel methods able to provide a fast response and high sensitivity comparable to the iQcheck and AFNOR validated methods for real-time diagnosis of contamination in foods [3,4].

Food processors need inexpensive, fast, and reproducible methods to be conducted by technicians on-site with easiness of testing. No system has been developed to meet all these criteria, while other non-biosensor technologies (such as lateral flow immunodetection) have achieved, in part, some of these requirements.

There is a need to distinguish between viable and non-viable pathogens, and this has been a topic well addressed by PCR analysis using DNA staining permeable to dead cells. Standard microbiology methods are based on culturing viable bacteria, therefore, they require three to four days for obtaining the results. While biosensor methods cannot distinguish dead bacteria, the time for biosensing analysis is very short, and there is the possibility to take action in the food distribution line when an alarm of contamination has been set. Food processing plants need to prevent any risk of accidental contamination of processing lines, and pathogens may be propagated in the food plants. Food processing plants are interested in assessing the completeness of their hygiene/cleaning efforts. These efforts do not involve the complexity of detection in foodstuffs, that is performed at microbiology laboratories, and this translates to a need to develop on-site bioensing systems for fast and accurate pathogen sensing.

In this review, we will introduce fluorescence-free biosensor methods applied to detection of pathogens contaminating food products, with a special focus on *L. monocytogenes* [6,7,8]. 

Biosensors often rely on direct detection methods, such as surface enhanced raman scattering (SERS), electrochemical methods, lateral flow immuno-assays, and on applications of metal nanoparticles, quantum dots, and nanomaterials for surface modification [9,10,11,12,13,14,15,16,17,18,19,20,21,22,23]. Among the detection methods providing signal enhancements at low bacteria contamination, the electrochemical methods, in which signal amplification is achieved through enzymes and redox cycling, have been recently extensively reviewed [17,18,19,20]. 

In this review, we will focus on surface plasmon resonance (SPR) and on SPR combined with immunoseparation from pre-enrichment broths [1,2,3,8] and on electrochemical impedance spectroscopy (EIS).

## 2. SPR Methods

The system of optical approach based on surface plasmon resonance (SPR) is performed with a light source and thin metallic material (Au). SPR is an optical technique that uses the evanescent wave produced by an incident, monochromatic light beam. The light beam interacts with free electrons (plasmons) in the metal film at the α angle (SPR angle) of incident light. The angle is dependent on the metal-dielectric interface. The prism based Kretschmann configuration is based on the excitation of a surface-bound electromagnetic wave from the metal side. The binding event between the investigated antigen and the capture antibody is recorded through the detection of a shift in the reflected light toward higher values of the SPR angle, or through a change in reflectance at a fixed angle, measuring changes in refractive index close to the sensor surface. SPR, exploiting as capture ligands either species-specific antibodies or nucleic acid aptamers, has been extensively applied to pathogen detection. 

However, using SPR methods, the results have been often limited to clear solutions with inoculated bacteria, and the LOD has been often too high and unsatisfactory for food safety applications. In various reports, SPR immunosensor measurements detected *L. monocytogenes and Salmonella* spp. cell suspensions at a concentration of 3–4 log CFU/mL [7,24,25,26,27].

In addition, low analytical sensitivity is either the result of a small refractive index, slow diffusion driven mass transfer, or the insufficient depth of the influenced layer. These are intrinsic problems in the conventional SPR methods. For the extension of SPR applications to food safety, either new surfaces [28,29,30] have been tested or new portable instruments have been developed [31].

### 2.1. SPR Imaging in Multiplex: Multichannel SPR Biosensors

SPR methods are able to evaluate bacterial presence in multiplex. Grating-coupled surface plasmon resonance imaging (GCSPRI) has been applied to multiplexed detection of microbes, toxins and viruses. In GCSPRI, disposable grating systems are obtained by deposition of thick metal films. Then, the wave is excited from the transparent material side: the sample is placed on a topographically-located position on the surface [32,33,34]. This system does not require a prism. GCSPRI was able to measure at the same time the binding of multiple regions of interest (ROIs) through an array of specific capture molecules immobilized on the surface.

#### Enhancement of Sensitivity by Combining SPR with a Labeling or Capturing Method

Standard SPR detection has been performed at relatively high concentrations of bacteria, often leading to non-specific binding. To circumvent this limitation, improvement of the SPR detection power has been achieved with an implementation of the technique. The sensitivity was significantly increased through the addition of antibody-nanoparticle conjugates (gold NP), as signal enhancers exerting a mass effect. The enhancement of the signal detecting the target antigens captured on a gold surface [35] has been applied also to other detection methods based on capture antibodies bound to a gold surface, such as the quarz crystal microbalance (QCM) [35].

As reported previously, antibody-functionalized gold nanoparticles (immuno-AuNP) have been added to bacteria captured on the surface, to enhance the reflectance units from the baseline, obtaining an increase in the SPR signal, with a LOD at an *L. monocytogenes* concentration of 2 log CFU/mL [1].

Localized surface plasmon resonance (LSPR) is an indirect method able to enhance SPR sensitivity [36,37,38]. The LSPR approach has been applied to label-free, real-time pathogen detection, and small, cost-effective LSPR biosensor systems have been constructed. Nevertheless, LSPR poses problems similar to SPR, such as a rapid decay of surface plasmon. Through the use of small binders, such as the fragment antigen-binding (Fab) portion of antibodies and aptamers, and by surface modification of the nanoparticles, an enhanced signal has been obtained.

Long-range SPR was applied in combination with magnetic nanoparticles and Au nanoparticles. In pathogen detection, the SPR propagated along a thin metal film, embedded in a symmetrical layer architecture with optimized refractive index, resulting in a dark-field light-scattering image, with detection achieved within 30 min [23].

### 2.2. EIS

Electrical biosensors measure the current and/or voltage changes following the binding of the antigens [39,40,41]. Electrical biosensors are grouped depending on the method used for electrical measuring, including voltammetry, amperometry/coulometry, and impedance. Among these, impedance sensing records the electrical impedance of an interface in alternate current (AC) steady state with constant bias conditions of direct current (DC). In the majority of experiments, the measure is obtained through the application of a weak sinusoidal voltage at a certain frequency allowing the evaluation of the current produced; the measure is then recorded at different frequencies. The measure of impedance is obtained by the current-voltage ratio. Electrochemical impedance spectroscopy (EIS) has been extensively applied to studies of a wide range of electrochemical measurements [42]. When the impedance of the electrode-solution interface varies after the target analyte binding to the antibody, EIS sensing records that impedance change. The other possible method is to evaluate the impedance or capacitance at a single frequency. Impedance measurement does not need additional reagents and allows label-free sensing. Impedance biosensing allows low cost, low power, multiplexing and miniaturization of the system, making them suitable for point-of-care (PoC) diagnostics and in detection of biological agents. 

#### 2.2.1. Fabrication of Impedance Sensor

Impedance biosensor fabrication is based on immobilization of the biorecognition probe onto a conductive electrode that allows to measure the interfacial impedance change when the analyte interacts with the probe. Biorecognition molecules are either antibodies, receptor proteins, peptides, or nuclei acid aptamers. Impedance biosensors can detect a large range of target analytes by changing the probe/biorecognition pairs. This makes impedance biosensors ideal for detection of: *L. monocytogenes* [43], allergens in food [44], environmental contaminants and endocrine disrupting chemicals (EDCs) [45].

The selection of electrode materials is of the utmost importance to the optimization of biosensor performance. An optimal surface allows to achieve its functionalization with relative simplicity. For electrochemical biosensors, the electrode materials are constrained by the needs of high electrical conductivity and the use of biocompatible materials. Biomolecules can denature when exposed to a metal surface for prolonged times. This has been circumvented by using gold, platinum and carbon as electrode materials in electrochemical biosensing [46]. Although biomolecules immobilization onto carbon electrodes can provide optimum stability [47,48], this material exhibits complex electrochemistry, that mainly depends on type of carbon, surface preparation and chemical treatment [49]. Si was also extensively used as a biosensor material. However, even if various combinations of metal surfaces and organic molecules have been applied to impedance sensing, noble metal surfaces (especially Au) have shown the best performances in fabrication and functionalization of interfaces for biosensing.

#### 2.2.2. Linkage of Bio-Molecules onto Au Surface

**Figure 1:**
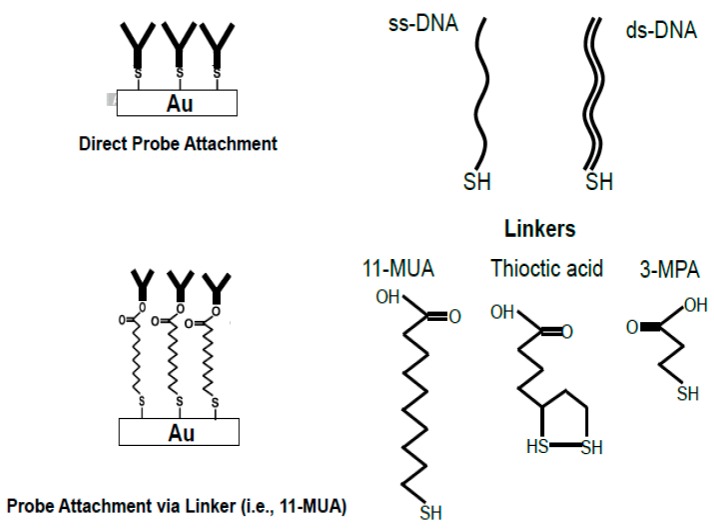
Direct and indirect attachment of molecules onto gold surfaces.

Two methods are mostly used to attach recognition probes onto a gold surface. The first is the direct attachment method, where the biomolecules are chemically modified, such that they have -SH groups at their terminals. One example of an attachment reaction is shown in Figure 1. The molecules directly attach to the gold surface through hydrophobic and electrostatic interactions [50]. However, this method is not suitable for EIS based sensing due to several reasons.

The surface coverage might be low. This is especially true for proteins and antibodies, which differ in shape, size, and orientation [51]. Direct attachment is more suitable for molecules, such as DNA, which have a well-defined structure [52]. It is challenging to block the active surface sufficiently. Since the molecules can be randomly arranged over the gold surface, it is difficult to avoid non-specific adsorption of interfering molecules [51]. Proteins adsorption onto the gold surface can be reversible. The proteins can be easily removed from the surface using certain solvents such as acetone and detergents such as Tween [53].

One way to overcome this problem is to use linker molecules. Functional alkanethiols serve as suitable linkers. Sulphur compounds (e.g., containing thiol groups) have a strong affinity to gold surfaces [54]. The gold-sulphur attachment occurs due to the oxidative addition of -SH bond to the gold surface. The bonding of the -SH group to Au is very strong [54]. 

Figure 1 shows three functional alkanethiols (11-mercaptoundeacanoic acid or 11-MUA, 3-mercaptoporpionic acid or MPA, and thioctic acid), having -SH groups and a carboxy-terminal COOH group. The COOH group is exploited to form covalent bonds with molecules provided with N-hydroxysuccinimide (NHS) esters, using the N-Ethyl-N′-(3-dimethylaminopropyl) carbodiimide hydrochloride (EDC)/NHS protocol [53]. This allows the stability of the capture probe during the time. In addition, the alkanethiols form self-assembled monolayers (SAM) with well-defined structure and thickness [54].

#### 2.2.3. Attaching Bio-Molecules

To covalently attach bio-molecules (probes) such as antibodies and proteins, NHS/EDC coupling chemistry has been most commonly used to form amine-reactive sites on functionalized gold or Si electrodes. These amine-reactive sites are subsequently exposed to probes [55], resulting in protein covalently bound to the surface. The majority of published studies have described detection of highly pure target proteins of interest, mostly on bacteria diluted in buffer or in a simple food matrix, while in other cases, authors have used real food matrices. The next challenges are to overcome poor reproducibility, non-specific binding, and the application of EIS to the analysis of clinical samples and complex food matrices. 

#### 2.2.4. Technical Challenges for Impedance Biosensors

Although impedance biosensors have been studied in many laboratories [46,47,48], there are several technical limitations. Among them, are: (a) nonspecific adsorption of compounds from complex matrices; (b) stability and reproducibility of probe immobilization onto a conductive electrode surface; and (c) complexity of impedance detection. 

##### Susceptibility to Non-Specific Adsorption

The most frequent criticism regarding impedance biosensors is that the method is susceptible to interference arising from non-specific adsorption [56,57,58]. Non-specific adsorption is ascribed to proteins contained in a complex matrix binding to the sensor interface not dependent on the recognition of the analyte by the probe. Non-specific adsorption is the cause of spurious signals during biosensing. Therefore, non-specific adsorption needs to be studied by control experiments using either complex test matrices or mixtures of different proteins or analytes.

Various solutions have been employed to overcome the problem, among them are sample dilution [59,60], adsorption of bovine serum albumin (BSA) [58] or other blocking reagents, and the use of a control electrode, devoid of the probe [61]. The use of sample dilution depends on the scope of the application and the detection limit to be obtained. When a monoclonal antibody is used for biomolecular recognition, a control electrode can be used with another antibody from the same animal whose antigen is unlikely to be found in the text matrix of interest. Recently, Suni et al., reported impedance detection of *L. monocytogenes* in tomato pulp with limit of detection of 4 CFU/mL [43], showing that non-specific adsorption was not detected neither detectable. Non-specific adsorption was determined through the comparison (ratio) of the impedance change at the measurement electrode (mouse monoclonal IgG_1_ antibody to *L. monocytogenes*) to that at a control electrode (mouse monoclonal IgG_1_ antibody to GAPDH). The method requires availability of adequate control electrodes, directed towards antigen targets not present in the samples to be tested. It was shown that multiple capture antibodies with different binding epitopes, and multiple control antibodies, increased the efficiency and efficacy to overcome non-specific binding.

##### Stability of Biomolecule Immobilization onto a Conductive Electrode Material

In impedance biosensor preparation, the immobilization of biomolecules onto a conductive and biocompatible electrode material is obtained in many cases by Au-thiol self-assembly chemistry [62]. The problem of stability of Au-thiol self-assembly chemistry is a factor limiting impedance biosensors application [63]. The present shelf-life is limited to days to weeks and depends on the storage conditions. A chemistry for biomolecule immobilization producing stability over time is also required for sensor calibration, and can involve use of aggressive chemicals. 

**Figure 2:**
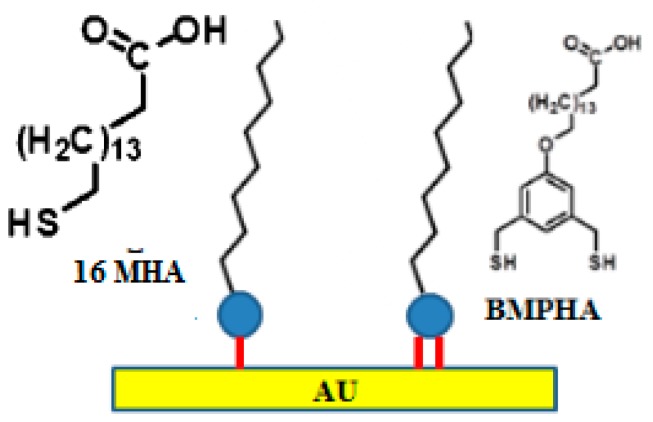
Structures of two carboxylic acid terminated alkanethiols.

A significant increase in stability can be achieved using multidentate thiols relative to monodentate thiols [64,65]. In the application of these reagents, multidentate alkanethiols showed able to produce SAMs either on flat as well as on curved gold surfaces at room temperature, showing an enhanced ability to withstand exposure to high temperatures in thermal desorption studies. The principle at the basis of their stability is the chelate effect, i.e., the free energy of the entropically favored bidendate binding, being two-times higher than that of monodendate binding [66]. Prof. T. Randall Lee’s research group at the University of Houston recently reported SAM formation on Au from the bidendate thiol 16-[3,5-bis(mercaptomethyl)phenoxy]-hexadecanoic acid (BMPHA) [44]. Following their results, Radhakrishnan and colleagues recently characterized the carboxylic acid-terminated alkanethiol, BMPHA, to produce highly stable carboxylic acid-terminated organic thin films [67]. In order to perform a complete analysis of the effectiveness of multidentate thiols, they fabricated and compared this SAM against the monothiol 16-mercaptohexadecanoic acid (16-MHA) based SAM. The detection limit for *Ara h* 1 allergen using the BMPHA linker was approximately 0.71 ng/mL (0.01 nM), which is about 10× lower than that obtained using the monodendate thiol, 16-mercaptohexadecanoic acid (16 MHA). Structures of both adsorbates are shown in Figure 2. 

Other types of substrates used for electrode fabrication have been based on carbon [68,69], Si [70,71], Pt [72,73], Ti [74,75], and indium tin oxide (ITO) [76,77]. In a recent report, degenerate (highly doped) Si was used as electrode material in EIS [78]. Degenerate Si has very weak electrical conductivity, and prevents the formation of a space charge layer during AC interrogation of the sensor interface. Radhakrishnan and Suni illustrated results demonstrating the possibility to regenerate the Si electrode during experiments during a 30-days storage using KSCN based solution [79]. This result showed the possible application and reusability of antibody-coated degenerate Si electrodes for EIS measurements, just performing the calibration on the day of use of electrodes. 

##### Complexity of Impedance Detection

Notwithstanding the substantial advances made in EIS based sensors, several obstacles and problems remain to be solved before their use in on-site detection. Basically, EIS sensor analysis needs operator skills in data processing. Costly impedance analyzers are needed when high test frequencies (>1 MHz) are applied. In addition, in some cases, impedance assays have been performed with already-processed samples. To be applicable as an on-site detection system, sample preparation must be simple and performable on site. Furthermore, the major part of currently tested systems requires at least 30 min for a single assay on abundant molecules and even longer times for highly diluted analytes. Even if EIS detection shows better performances compared to the standard enzyme-linked immunosorbent assay (ELISA) method, the incubation time may be reduced further. The assay time could be improved through the incorporation of the AC electrokinetic (ACEK) microfluidics platform [80,81,82,83,84,85], established in the 1990s, into the impedance sensor. The ACEK effects have been studied as a means to manipulate particles or macromolecules. ACEK effects is based on an AC electric field that induce particle and fluid movement [85]. As shown by several researchers [81,82,83,84], ACEK in combination with microelectrodes can induce in situ concentration of particles to improve detection sensitivity and throughput. ACEK, as a particle and fluid manipulation system, has low requirements for device fabrication and operation, so that it can be incorporated into a detection system, by addition of microelectrodes and their AC signal source. When a non-homogeneous AC electric field is applied to an aqueous solution, particle movement and microflows are induced in order to transport particles. Direct particle movement is generated by dielectrophoresis (DEP), while particles can also be moved by microflows such as AC electroosmosis or AC electrothermal flows, making them to reach the microsensor. The movement of particles by DEP is based on the difference between the polarizability of the particles and that of the solution, at a certain frequency. An AC electric field generates microflows through AC electroosmosis (ACEO) that is prevalent at low ionic strengths. The flow velocity of ACEO has been shown to decrease significantly with increasing conductivity, and dropping to zero above 0.085 S m^−1^ [86,87]. Most medical and biological applications involve the use of solution with high conductivity, so the ACEO flow will be negligible. The AC electric field generates microflows through ACET effect, due to non-homogeneous heating of electrolytic fluids by the passage of electric current. Thus, the ACET flow is almost frequency-independent, scaling up with the electrical conductivity of fluid. Using planar electrodes, the ACET effect induces vortices above each electrode, and the microflows convect the embedded particles towards the electrode surface [86]. Since fluidic forces are not dependent on particle size, ACET microflows are adapted to transport macromolecules to the electrodes. In a recent report, Liu and colleagues showed the ACET effect has an important role in increasing detection sensitivity [87]. Thus, through the combination of DEP and ACET effects, the ACEK-based impedance sensor has been shown capable and effective to enrich the concentration of nanoscale particles over a wide range of values, further reducing the incubation time and thus realizing a rapid detection system.

### 2.3. Further Advancements and Present Perspectives

EIS methods have been often applied successfully to detects in real samples, such as plant extracts and sera [88,89]. Validation efforts are being conducted to demonstrate the robustness of detection, reproducibility and variability in multi-site installations. Previously protein chips were shown to provide an accurate detection method, showing good sensitivity and reproducibility [1,2,3,8]. A comparative study of pathogen detection with protein chip and Real Time PCR performed on the same samples has shown the possibility to detect *L. monocytogenes* with good sensitivity and reproducibility, with the lowest LOD 2.55 CFU/mL, achieved by PCR [3]. However, the manufacture of complex detection systems such as protein chips introduces several variables, influenced by a series of cooperative factors (spots variability, local surface variability, different degree of blocking efficiency, background noise and signal to noise ration variability) affecting experiment reproducibility. Therefore, fluorescence-free biosensors can overcome these bottlenecks and perform in a more reproducible way than protein chip methods.

The application of biosensing methods, such as enhanced SPR and EIS biochips are fluorescent-free, easy to operate, fast and low-cost methods, feasible to perform multiple detection and on-site assays showing high sensitivity [88,89]. Particular interest has been focused on the development of lab-on-chip (LOC) technologies for the ease of the handling and operational requirements, reduction of volumes through miniaturization, higher surface-to-volume ratio in microchannels and application of microfluidics, with the possibility to integrate additional components (micropillars, micropores, microfilters, mixers) [6]. An interesting result was achieved by means of recording of displacement of magnetic bead-bound bacterial clusters using periodic magnetic fields at a given frequency, allowing to reach a sensitive detection of bacteria, from 10^2^ cells·mL^−1^ to very few bacteria [90]. A recently described EIS protocol has made possible the detection of *L. monocytogenes* with LOD as low as 1 log CFU/mL [90] and the extension of the applicability to multiplex analyses [91]. In the field of SPR, concomitant “on-chip” microbial culture with sensitive SPR detection was achieved exploiting a lab-on-chip platform [92]. In addition, using a thin chip layer based on Co-Au alloy, authors reported about the potential of magneto optical surface plasmon resonance (MOSPR) assay in detection of analytes [93]. Further studies are required to show the reproducibility and robustness of the method, even in the presence of different food matrices.

## 3. Conclusions

SPR and EIS biosensors and similar biosensing tools are under development for the on-site, early and multiplexed quantitative detection of pathogenic bacteria, for food diagnostics and biosecurity purposes. Further advancements are envisaged through the application of superior surface modification methods, such as the use of bidendate thiol 16-[3,5-bis(mercaptomethyl)phenoxy]-hexadecanoic acid (BMPHA), the application of proper solutions to stabilize the capture ligands, and the application of ACEK microflow mechanisms, such as AC electroosmosis (ACEO) and AC electrothermal (ACET) effects. Furthermore, an increase in the limit of detection may be achieved by combining the biosensor methods with immunoseparation of bacteria from larger volumes. It is envisaged that improved biosensing methods can respond to food safety issues in the shortest time possible and provide safety certification to the food chain even at retailer shops and refectory level.

## References

[1] Poltronieri P., De Blasi M.D., D’Urso O.F. (2009). Detection of *Listeria monocytogenes* through Real Time PCR and biosensor methods. Plant Soil Environ..

[2] Poltronieri P., Cimaglia F., De Lorenzis E., Chiesa M., Mezzolla V., Reca I.B. (2016). Protein chips for detection of *Salmonella* spp. from enrichment culture. Sensors.

[3] Cimaglia F., De Lorenzis E., Mezzolla V., Rossi F., Poltronieri P. (2016). Detection of *L. monocytogenes* in enrichment cultures by immunoseparation and immunosensors. IEEE Sens..

[4] Rodriguez-Lazaro D., Gonzalez-García P., Gattuso A., Gianfranceschi M.V., Hernandez M. (2014). Reducing time in the analysis of *Listeria monocytogenes* in meat, dairy and vegetable products. Int. J. Food Microbiol..

[5] Lauer W.F., Sidi C.D., Tourniaire J.P. (2009). iQ-Check Salmonella II: Real-time polymerase chain reaction test kit. Performance tested method 010803. J. AOAC Int..

[6] Poltronieri P., Mezzolla V., Primiceri E., Maruccio G. (2014). Biosensors for detection of food pathogens. Foods.

[7] Poltronieri P. (2016). Innovations in detection of deliberate or accidental contamination with biological agents in environment and foods. Challenges.

[8] D’Urso O.F., De Blasi M.D., Manera M.G., Latronico M.F., Rella R., Poltronieri P. (2008). *Listeria monocytogenes* detection with surface plasmon resonance and protein arrays. IEEE Sens..

[9] Menti C., Henriques J.A.P., Missell F.P., Roesch-Ely M. (2016). Antibody-based magneto-elastic biosensors: Potential devices for detection of pathogens and associated toxins. Appl. Microbiol. Biotechnol..

[10] Rippa M., Castagna R., Pannico M., Musto P., Borriello G., Paradiso R., Galiero G., Bolletti Censi S., Zhou J., Zyss J. (2017). Octupolar metastructures for a highly sensitive, rapid, and reproducible phage-based detection of bacterial pathogens by Surface-Enhanced Raman Scattering. ACS Sens..

[11] Juan-Colás J., Johnson S., Krauss T.F. (2017). Dual-mode Electro-Optical techniques for biosensing applications: A Review. Sensors.

[12] Byrne B., Stack E., Gilmartin N., O’Kennedy R. (2009). Antibody-based sensors: Principles, problems and potential for detection of pathogens and associated toxins. Sensors.

[13] Wang H., Li Y., Wang A., Slavik M. (2011). Rapid, sensitive, and simultaneous detection of three foodborne pathogens using magnetic nanobead-based immunoseparation and quantum dot-based multiplex immunoassay. J. Food Prot..

[14] Huang X., Xu Z., Mao Y., Ji Y., Xu H., Xiong Y., Li Y. (2015). Gold nanoparticle-based dynamic light scattering immunoassay for ultrasensitive detection of *Listeria monocytogenes* in lettuces. Biosens. Bioelectron..

[15] Sarkar D., Gunda N.S., Jamal I., Mitra K. (2014). Optical biosensors with an integrated Mach-Zender interferometer for detection of *Listeria monocytogenes*. Biomed. Microdevices.

[16] Ohk S.H., Bhunia A.K. (2013). Multiplex fiber optic biosensor for detection of *Listeria monocytogenes*, *Escherichia coli* O157:H7 and *Salmonella enterica* from ready-to-eat meat samples. Food Microbiol..

[17] Piro B., Reisberg S. (2017). Recent advances in electrochemical immunosensors. Sensors.

[18] Cinti S., Volpe G., Piermarini S., Delibato E., Palleschi G. (2017). Electrochemical biosensors for rapid detection of foodborne Salmonella: A critical overview. Sensors.

[19] Campuzano S., Yáez-Sedeño P., Pingarrón J.M. (2017). Electrochemical affinity biosensors in food safety. Chemosensors.

[20] Kokkinos C., Economou A., Prodromidis M.I. (2016). Electrochemical immunosensors: Critical survey of different architectures and transduction strategies. TrAC Trends Anal. Chem..

[21] Cimaglia F., Rosu V., Chiesa M., Poltronieri P., Aliverti A., Santino A., Sechi L.A. (2012). Quantum dot nanoparticle-based lateral flow assay for rapid detection of *Mycobacterium* species using anti-FprA antibodies. Nanotechnol. Dev..

[22] Wang Y., Li H., Wang Y., Li H., Luo L., Xu J., Ye C. (2017). Development of multiple cross displacement amplification label-based gold nanoparticles lateral flow biosensor for detection of *Listeria monocytogenes*. Int. J. Nanomed..

[23] Cho I.-H., Ku S. (2017). Current technical approaches for the early detection of foodborne pathogens: Challenges and opportunities. Int. J. Mol. Sci..

[24] De Lorenzis E., Manera M.G., Cimaglia F., Montagna G., Chiesa M., Poltronieri P., Santino A., Rella R. (2013). SPR based immunosensor for detection of *Legionella pneumophila* in water samples. Opt. Commun..

[25] Nanduri V., Bhunia A.K., Tu S.-I., Paoli G.C., Brewster J.D. (2007). SPR biosensor for the detection of *L. monocytogenes* using phage-displayed antibody. Biosens. Bioelectron..

[26] Komatsu H., Miyachi M., Fujii E., Citterio D., Yamada K., Sato Y., Kurihara K., Kawaguchi H., Suzuki K. (2006). SPR sensor signal amplification based on dye-doped polymer particles. Sci. Technol. Adv. Mater..

[27] Liu X., Hu Y., Zheng S., Liu Y., He Z., Luo F. (2016). Surface plasmon resonance immunosensor for fast, highly sensitive, and in situ detection of the magnetic nanoparticles-enriched *Salmonella enteritidis*. Sens. Actuators B Chem..

[28] Wang Y., Ye Z., Si C., Ying Y. (2013). Monitoring of *Escherichia coli* O157:H7 in food samples using lectin based surface plasmon resonance biosensor. Food Chem..

[29] Wang X., Zhan S., Huang Z., Hong X. (2013). Review: Advances and applications of Surface Plasmon Resonance biosensing instrumentation. Instrum. Sci. Technol..

[30] Oliverio M., Perotto S., Messina G.C., Lovato L., De Angelis F. (2017). Chemical functionalization of plasmonic surface biosensors: A tutorial review on issues, strategies, and costs. ACS Appl. Mater. Interfaces.

[31] Wang S., Xie J., Jiang M., Chang K., Chen R., Ma L., Zhu J., Guo Q., Sun H., Hu J. (2016). The development of a portable SPR bioanalyzer for sensitive detection of *Escherichia coli* O157:H7. Sensors.

[32] Zhang X., Tsuji S., Kitaoka H., Kobayashi H., Tamai M., Honjoh K.-I., Miyamoto T. (2017). Simultaneous detection of *Escherichia coli* O157:H7, *Salmonella enteritidis*, and *Listeria monocytogenes* at a very low level using Simultaneous Enrichment Broth and multichannel SPR biosensor. J. Food Sci..

[33] Marusov G., Sweatt A., Pietrosimone K., Benson D., Geary S.J., Silbart L.K., Challa S., Lagoy J., Lawrence D.A., Lynes M.A. (2012). A microarray biosensor for multiplexed detection of microbes using grating-coupled surface plasmon resonance imaging. Environ. Sci. Technol..

[34] Vaisocherová-Lísalová H., Víšová I., Ermini M.L., Špringer T., Chadtová Song X., Mrázek J., Lamačová J., Lynn N.S., Šedivák P., Homola J. (2016). Low-fouling surface plasmon resonance biosensor for multi-step detection of foodborne bacterial pathogens in complex food samples. Biosens. Bioelectron..

[35] Guo X., Lin C.S., Chen S.H., Ye R., Wu V.C. (2012). A piezoelectric immunosensor for specific capture and enrichment of viable pathogens by quartz crystal microbalance sensor, followed by detection with antibody-functionalized gold nanoparticles. Biosens. Bioelectron..

[36] Unser S., Bruzas I., He J., Sagle L. (2015). Localized Surface Plasmon Resonance biosensing: Current challenges and approaches. Sensors.

[37] Song L., Zhang L., Huang Y., Chen L., Zhang G., Shen Z., Zhang J., Xiao Z., Chen T. (2017). Amplifying the signal of localized surface plasmon resonance sensing for the sensitive detection of *Escherichia coli* O157:H7. Sci. Rep..

[38] Rippa M., Castagna R., Tkachenko V., Zhou J., Petti L. (2017). Engineered nanopatterned substrates for high-sensitive localized surface plasmon resonance: An assay on biomacromolecules. J. Mater. Chem. B.

[39] Thévenot D.R., Toth K., Durst R.A., Wilson G.S. (2001). Electrochemical biosensors: Recommended definitions and classification. Biosens. Bioelectron..

[40] Drummond T.G., Hill M.G., Barton J.K. (2003). Electrochemical DNA sensors. Nat. Biotechnol..

[41] Bakker E. (2004). Electrochemical sensors. Anal. Chem..

[42] Macdonald J.R. (1987). Impedance Spectroscopy: Emphasizing Solid Materials and Systems.

[43] Radhakrishnan R., Jahne M., Rogers S., Suni I.I. (2013). Detection of *Listeria monocytogenes* by Electrochemical Impedance Spectroscopy. Electroanalysis.

[44] Radhakrishnan R., Pali M., Lee H.J., Lee T.R., Suni I.I. (2016). Impedance biosensor incorporating a Carboxylate-Terminated Bidentate Thiol for antibody immobilization. J. Electrochem. Soc..

[45] Bever C.R.S., Majkova Z., Radhakrishnan R., Suni I.I., McCoy M., Wang Y., Dechant J., Gee S., Hammock B.D. (2014). Development and utilization of camelid VHH antibodies from Alpaca for 2,2′4,4′-tetrabrominated diphenyl ether detection. Anal. Chem..

[46] Katz E., Willner I. (2003). Probing biomolecular interactions at conductive and semiconductive surfaces by impedance spectroscopy: Routes to impedimetric immunosensors, DNA sensors and enzyme biosensors. Electroanalysis.

[47] Blankespoor R., Limoges B., Schöllhorn B., Syssa-Magalé J.L., Yazidi D. (2005). Dense monolayers of metal-chelating ligands covalently attached to carbon electrodes electrochemically and their useful application in affinity binding of histidine-tagged proteins. Langmuir.

[48] Teh H.F., Gong H., Dong X.-D., Zeng X., Kuan Tan A.L., Yang X., Tan S.N. (2005). Electrochemical biosensing of DNA with capture probe covalently immobilized onto glassy carbon surface. Anal. Chim. Acta.

[49] Ramesh P., Sampath S. (2003). Electrochemical characterization of binderless, recompressed exfoliated graphite electrodes: Electron transfer kinetics and diffusion characteristics. Anal. Chem..

[50] Maupas H., Soldatkin A.P., Martelet C., Jaffrezic-Renault N., Mandrand B. (1997). Direct immunosensing using differential electrochemical measurements of impedimetric variations. J. Electroanal. Chem..

[51] Rickert J., Göpel W., Beck W., Jung G., Heiduschka P. (1996). A mixed self assembled monolayer for an impedimetric immunosensors. Biosens. Bioelectron..

[52] Steel A.B., Levicky R.L., Herne T.M., Tarlov M.J. (2000). Immobilization of nucleic acids at solid surfaces: Effect of oligonucleotide length on layer assembly. Biophys. J..

[53] Patel N., Davies M.C., Hartshorne M., Heaton R.J., Roberts C.J., Tendler S.J.B., Williams P.M. (1997). Immobilization of protein molecules onto homogeneous and mixed carboxylate-terminated self assembled monolayers. Langmuir.

[54] Ulman A. (1996). Formation and structure of self assembled monolayers. Chem. Rev..

[55] Homola J. (2008). Surface Plasmon resonance sensors for detection of chemical and biological species. Chem. Rev..

[56] Ostuni E., Chapman R.G., Holmlin R.E., Takayama S., Whitesides G.M. (2001). A survey of structure-property relationships of surfaces that resist the adsorption of protein. Langmuir.

[57] Bange A., Halsall H.B., Heineman W.R. (2005). Microfluidic immunosensor systems. Biosens. Bioelectron..

[58] Shankaran D.R., Gobi V.K., Miura N. (2007). Recent advancements in surface plasmon resonance immunosensors for detection of small molecules of biomedical, food and environmental interest. Sens. Actuators B.

[59] Gaus K., Hall E.A. (1998). Surface Plasmon resonance sensor for heparin measurements in blood plasma. Biosens. Bioelectron..

[60] Andersson L.I., Hardenborg E., Sandberg-Stall M., Moller K., Henriksson J., Bramsby-Sjostrom I., Olsson L.I., Abdel-Rahim M. (2004). Development of a molecularly imprinted polymer based solid-phase extraction of local anaesthetics from human plasma. Anal. Chim. Acta.

[61] Singh R., Suni I.I. (2010). Minimizing non-specific adsorption in protein biosensors that utilize electrochemical impedance spectroscopy. J. Electrochem. Soc..

[62] Love J.C., Estroff L.A., Kriebel J.K., Nuzzo R.G., Whitesides G.M. (2005). Self-assembled monolayers of thiolates on metals as a form of nanotechnology. Chem. Rev..

[63] Srimsobat L., Jamison A.C., Lee T.R. (2011). Stability: A key issue for self-assembled monolayers on gold as thin film coatings and nanoparticle protectants. Colloid Surf. A.

[64] Srisombat L., Zhang S., Lee T.R. (2010). Thermal stability of Mono-, Bis-, and Tris-chelating alkanethiol films assembled on gold nanoparticles and evaporated flat gold. Langmuir.

[65] Ge D., Wang X., Williams K., Levicky R. (2012). Thermostable DNA immobilization and temperature effects on surface hybridization. Langmuir.

[66] Chinwangso P., Jamison A.C., Randall Lee T. (2011). Multidendate adsorbates for self assembled monolayer films. Acc. Chem. Res..

[67] Lee H.J., Jamison A.C., Yuan Y., Li C.-H., Rittikulsittichai S., Rusakova I., Randall Lee T. (2013). Robust carboxylic acid terminated organic thin films and nanoparticle protectants generated from bidendate alkanethiols. Langmuir.

[68] Brett C.M.A., Oliveira-Brett A.M., Serrano S.H.P. (1999). An EIS study of DNA-modified electrodes. Electrochim. Acta.

[69] Davis F., Nabok A.V., Higson S.P. (2005). Species differentiation by DNA-modified carbon electrodes using AC impedimetric approach. Biosens. Bioelectron..

[70] Cai W., Peck J.R., van der Weide D.W., Hamers R.J. (2004). Direct electrical detection of hybridization at DNA-modified silicon surface. Biosens. Bioelectron..

[71] Yang W.S., Butler J.E., Russell J.N., Hamers R.J. (2007). Direct electrical detection of antibody-antigen binding on diamond and silicon substrates using electrical impedance spectroscopy. Analyst.

[72] De Silva M.S., Zhang Y., Hesketh P.J., Maclay G.J., Gendel S.M., Stetter J.R. (1995). Impedance based sensing of the of the specific binding reaction between *Staphylococcus enterotoxin B* and its antibody on an ultrathin Pt film. Biosens. Bioelectron..

[73] Pak S.C., Penrose W., Hesketh P.J. (2001). An ultrathin platinum film sensor to measure biomolecular binding. Biosens. Bioelectron..

[74] Mantzila A.G., Prodromidis M.I. (2005). Performance of impedimetric biosensors based on anodically formed Ti/TiO_2_ electrodes. Electroanalysis.

[75] Mantzila A.G., Prodromidis M.I. (2006). Development and study of anodic Ti/TiO_2_ electrodes and their potential use as impedimetric immunosensors. Electrochim. Acta.

[76] Ruan C.M., Yang L., Li Y.B. (2002). Immunobiosensor chips for detection of *Escherichia coli* O157:H57 using electrochemical impedance spectroscopy. Anal. Chem..

[77] Corry B., Janelle U., Crawley C. (2003). Probing direct binding affinity in electrochemical antibody-based sensors. Anal. Chim. Acta.

[78] Huang Y., Suni I.I. (2008). Degenerate Si as an electrode material for electrochemical biosensors. J. Electrochem. Soc..

[79] Radhakrishnan R., Suni I.I. (2016). Antibody regeneration on degenerate Si electrodes for calibration and reuse of impedance biosensors. Sens. Bio-Sens..

[80] Morgan H., Green N.G. (2003). AC Electrokinetics: Colloids and Nanoparticles.

[81] Wang D., Sigurdson M., Meinhart C.D. (2005). Experimental analysis of particle and fluid motion in AC electrokinetics. Exp. Fluids.

[82] Ahualli S., Jimenez M.L., Carrique F., Delgado A.V. (2009). AC electrokinetics of concentrated suspensions of soft particles. Langmuir.

[83] Wu J. (2006). Biased AC electro-osmosis for on-chip bioparticle processing. IEEE Trans. Nanotechnol..

[84] Wu J. (2008). Interactions of electrical fields with fluids: Laboratory-on-a-chip applications. IET Nanobiotechnol..

[85] Castellanos A., Ramos A., Gonzalez A., Green N.G., Morgan H. (2003). Electrohydrodynamics and dielectrophoresis in microsystems: Scaling laws. J. Phys. D Appl. Phys..

[86] Lian M., Islam N., Wu J. (2007). AC electrothermal manipulation of conductive fluids and particles for lab-chip applications. IET Nanobiotechnol..

[87] Liu X., Yang K., Wadhwa A., Eda S., Li S., Wu J. (2011). Development of an AC electrokinetics-based immunoassay system for on-site serodiagnosis of infectious diseases. Sens. Actuators A.

[88] Jarocka U., Wąsowicz M., Radecka H., Malinowski T., Michalczuk L., Radecki J. (2011). Impedimetric immunosensor for detection of plum pox virus in plant extracts. Electroanalysis.

[89] Jarocka U., Sawicka R., Góra-Sochacka A., Sirko A., Zagórski-Ostoja W., Radecki J., Radecka H. (2014). Immunosensor based on antibody binding fragments attached to gold nanoparticles for detection of avian influenza virus H5N1. Sensors.

[90] David S., Polonschii C., Gheorghiu M., Bratu D., Dobre A., Gheorghiu E. (2013). Assessment of pathogenic bacteria using periodic actuation. Lab Chip.

[91] Primiceri E., Chiriacò M.S., De Feo F., Santovito E., Fusco V., Maruccio G. (2016). A multipurpose biochip for food pathogen detection. Anal. Methods.

[92] Bouguelia S., Roupioz Y., Slimani S., Mondani L., Casabona M.G., Durmort C., Vernet T., Calemczuk R., Livache T. (2013). On-chip microbial culture for the specific detection of very low levels of bacteria. Lab Chip.

[93] David S., Polonschii C., Gheorghiu M., Bratu D., Gheorghiu E. (2017). Biosensing based on Magneto-Optical Surface Plasmon Resonance. Methods Mol. Biol..

